# 共价有机框架材料磁固相萃取-高效液相色谱法分析环境水体中对羟基苯甲酸酯

**DOI:** 10.3724/SP.J.1123.2022.06006

**Published:** 2022-11-08

**Authors:** Yue BAO, Yixin ZHAI, Tao NING, Pin CHEN, Shukui ZHU

**Affiliations:** 中国地质大学(武汉), 生物地质与环境地质国家重点实验室, 湖北 武汉 430074; State Key Laboratory of Biogeology and Environmental Geology, China University of Geosciences, Wuhan 430074, China

**Keywords:** 共价有机框架, 磁固相萃取, 高效液相色谱, 对羟基苯甲酸酯, 环境水样, covalent organic framework (COF), magnetic solid-phase extraction (MSPE), high performance liquid chromatographic (HPLC), parabens, environment water

## Abstract

建立了一种基于共价有机框架材料的磁固相萃取-高效液相色谱方法,用于环境水样中对羟基苯甲酸酯的快速灵敏分析。首先以Fe_3_O_4_纳米粒子为磁核,通过1,3,5-苯三甲醛(Tb)和联苯胺(Bd)在室温下的席夫碱反应合成了磁性共价有机框架材料——Fe_3_O_4_@TbBd,通过扫描电镜、热重分析、X射线衍射和振动样品磁强计等表征手段证明了该磁性共价有机框架材料具有良好的热稳定性和化学稳定性,且磁响应强度较大,是用于磁固相萃取的理想材料。随后系统研究了影响萃取效率的因素,包括吸附剂用量、萃取时间、pH、解吸溶剂、解吸时间和解吸次数,建立了基于Fe_3_O_4_@TbBd的磁固相萃取-高效液相色谱测定环境水样中4种对羟基苯甲酸酯的方法。方法的线性范围良好,4种目标物的检出限和定量限范围分别为0.2~0.4 μg/L和0.7~1.4 μg/L,加标回收率为86.1%~110.8%,日内和日间精密度的相对标准偏差(RSD)分别低于5.5%和4.9%。最后将该方法应用于东湖水、长江水和生活废水中对羟基苯甲酸酯的测定,不同加标水平下对羟基苯甲酸酯的回收率在80.7%~117.5%之间,RSD在0.2%~8.8%之间。该方法操作简单,萃取时间短,灵敏度较高且对环境友好,在环境水样中对羟基苯甲酸酯的检测方面有良好的应用潜力。

## Abstract (en)

Parabens are a class of antimicrobial preservatives that are widely used in cosmetics, pharmaceuticals, and food products because of their ease of production, antimicrobial effect, and low price. The widespread use of these parabens, poses potential risks to human health. Therefore, it is necessary to establish a simple and rapid method for the detection of parabens. The large number of substrate interferences in complex samples is an important factor affecting the sensitivity of analytical methods. Magnetic solid-phase extraction (MSPE) has received much attention because of its advantages of easy operation, short extraction time, small sample amount, low cost, and environmental friendliness. Covalent organic frameworks (COFs) with high crystallinity, high specific surface area, adjustable pore size, regular porosity, as well as high chemical and thermal stability are now widely used in separation and analysis. Therefore, a sample pretreatment method combining MSPE and COF for the analysis of parabens in complex matrices is very promising. A magnetic covalent organic framework, Fe_3_O_4_@TbBd, was successfully synthesized by the Schiff base reaction of 1,3,5-triformylbenzene (Tb) and benzidine (Bd) at room temperature using Fe_3_O_4_ nanoparticles as magnetic cores. Characterization by scanning electron microscopy (SEM), thermogravimetric analysis (TGA), X-ray diffraction (XRD), vibrating sample magnetometer (VSM) measurements, etc. revealed that the magnetic COF has high magnetic responsiveness, as well as good thermal and chemical stability, which make it an ideal adsorbent for the MSPE of parabens. Some factors related to the extraction efficiency, including the amount of adsorbent, extraction time, pH, desorption solvent, desorption time, and number of desorption were systematically investigated. A method involving MSPE and high performance liquid chromatography-ultraviolet detection (HPLC-UV) based on the Fe_3_O_4_@TbBd was developed for the determination of four parabens (ethylparaben, propylparaben, butylparaben, and benzylparaben) in environmental water samples. Under the optimal extraction conditions, the method showed good linearities. The limits of detection and limits of quantification were 0.2-0.4 μg/L and 0.7-1.4 μg/L for the four analytes, respectively. The recoveries at three spiked levels were in the range of 86.1%-110.8% with intra-day and inter-day RSDs of less than 5.5% and 4.9%, respectively. The method was successfully applied to the determination of parabens in East Lake water, Yangtze water, and domestic wastewater. Ethyl paraben and propyl paraben were detected in domestic wastewater at the levels of 1.8 μg/L and 0.4 μg/L, respectively. The recoveries of the parabens at different spiked levels ranged from 80.7% to 117.5%, with RSDs of 0.2%-8.8%. The method has good potential for the determination of parabens in environmental water samples because of its operational simplicity, short extraction time, high sensitivity, and environmental friendliness.

对羟基苯甲酸酯是一类抗菌防腐剂,通过破坏细胞膜和胞内蛋白质并改变微生物细胞酶活性,起到防腐的作用。对羟基苯甲酸酯具有易生产、抗菌效果好、价格便宜等优点,被广泛用于化妆品、药品和食品的防腐剂中^[1,2]^。

随着化妆品和药品使用的不断增加,环境中对羟基苯甲酸酯浓度也相应增加,例如在地表水中已有对羟基苯甲酸酯的检出记录^[3]^。同时,部分地方农业灌溉用水中对羟基苯甲酸酯的浓度较高,且对废水处理不当,导致土壤和地下水中出现对羟基苯甲酸酯的污染^[4,5]^。长期使用含对羟基苯甲酸酯防腐剂的化妆品会对人体皮肤产生潜在伤害,同时一些药品和保健品中添加的对羟基苯甲酸酯也可能进入到人体中,干扰内分泌系统的正常运行,例如可能导致女性乳腺癌发病率的增加^[6,7]^。因此,很多国家已经对这类新兴污染物制定了严格的监管政策,例如在中国和日本,环境水域中对羟基苯甲酸酯的最大含量限制分别为0.4%和1.0%^[8]^,欧盟规定了商业产品中每种对羟基苯甲酸酯的最大含量为0.4%,且对羟基苯甲酸酯的总含量不超过0.8%^[2]^。为了有效监管和控制对羟基苯甲酸酯的使用,建立复杂基质中对羟基苯甲酸酯的简单、快速、灵敏的样品前处理及分析方法尤为重要。

目前为止报道了多种样品前处理方法用于不同样品中对羟基苯甲酸酯的萃取,如固相萃取^[9]^、固相微萃取^[10]^、搅拌棒吸附萃取^[11]^和磁固相萃取(magnetic solid-phase extraction, MSPE)^[12]^。MSPE是在固相萃取基础上发展起来的一种新型样品前处理技术,具有操作简便、萃取时间短、样品用量小、有机溶剂用量少、成本低并且环境友好等优势^[13]^。磁性吸附剂的选择对MSPE萃取效率、富集因子、选择性和抗干扰能力的影响重大,是获得良好萃取性能的关键因素。目前越来越多不同种类的磁性吸附剂被开发并应用于MSPE中,如磁性纳米碳材料(石墨烯、氧化石墨烯、碳纳米管等)^[14,15]^、磁性分子印迹聚合物^[16]^、磁性金属有机框架^[17]^和磁性共价有机框架^[18]^,这些磁性吸附剂在环境、食品、生物等领域的应用都有着巨大的潜力。其中,共价有机框架(COF)是通过共价键将有机构建模块连接在一起而形成的一类晶体有机多孔结构,主要由轻元素(H、B、C、N和O)组成,呈现出二维或三维的拓扑结构^[19]^。COF具有独特的物理和化学特性:高结晶度、高比表面积、可调节的孔径、规则的孔隙率、高化学稳定性和热稳定性,在气体吸附与储存、多相催化、能量储存和污染物的去除、降解和分离等领域具有较好的应用前景^[20⇓⇓-23]^。此外,COF的可设计性和易于功能化等特点也可用于制备性能优异的磁性吸附剂,例如根据目标污染物的结构与特性来设计COF材料,构建出新型磁性纳米复合材料用于特定目标分子的萃取。

在本研究中,我们运用了一种绿色温和的方法,在室温条件下通过联苯胺和1,3,5-苯三甲醛之间的席夫碱反应成功合成了COF材料——TbBd,并将其负载在四氧化三铁纳米粒子上,建立了基于TbBd的磁固相萃取-高效液相色谱分析环境水样中4种对羟基苯甲酸酯的方法。

## 1 实验部分

### 1.1 试剂与仪器

六水氯化铁(FeCl_3_·6H_2_O)、柠檬酸钠(Na_3_C_6_H_5_O_7_·2H_2_O)、乙酸钠、盐酸、甲醇、乙腈购于上海国药集团化学试剂有限公司,均为分析纯;联苯胺(benzidine, Bd)购于河南阿尔法化工有限公司,纯度为95%; 1,3,5-苯三甲醛(1,3,5-triformylbenzene, Tb)、对羟基苯甲酸乙酯(ethylparaben, EtP)、对羟基苯甲酸丙酯(propylparaben, PrP)、对羟基苯甲酸丁酯(butylparaben, BuP)、对羟基苯甲酸苯甲酯(benzylparaben, BzP)均购于吉林中科研伸科技有限公司,Bd和EtP的纯度为98%, PrP和BuP的纯度为99%, BzP的纯度为96%。图1给出了4种对羟基苯甲酸酯的结构式、分子式和log *K*_ow_。

**图1:**
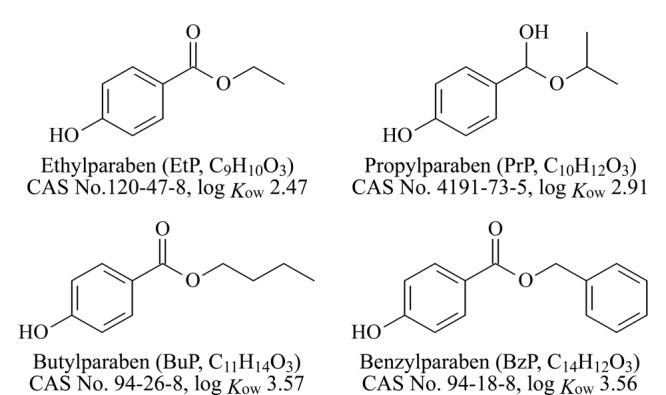
4种对羟基苯甲酸酯的结构和性质

LC-16 HPLC高效液相色谱仪和SPD-16 UV紫外检测器(日本岛津有限公司), Nicolet iS20傅里叶变换红外光谱仪(美国赛默飞世尔科技有限公司), Ultima IV X射线粉晶衍射仪(日本Rigaku公司), SU8010高分辨场发射扫描电镜仪(日本日立公司), 7404振动样品磁强计(美国LakeShore公司), TGA 550热重分析仪(美国TA公司), PS-80A超声波清洗仪(东莞市洁康超声波设备有限公司), 12N/A冷冻干燥机(宁波新芝生物科技股份有限公司), 18.2 MΩ·cm超纯水由超纯水恒力净水机(力康国际贸易(上海)有限公司)制得。

### 1.2 标准样品的配制

分别称取对羟基苯甲酸酯标准样品,溶解于甲醇中,配制成200 mg/L的单标准储备液,然后用甲醇稀释依次配制成20 mg/L和2 mg/L的单标准储备液。取一定量的单标准储备液依次配制成200、20和2 mg/L的4种对羟基苯甲酸酯的混合标准储备液。以上标液均在4 ℃冰箱中保存。

### 1.3 磁性COFs材料的合成

#### 1.3.1 Fe_3_O_4_纳米粒子(Fe_3_O_4_NPs)的合成

采用溶剂热法制备四氧化三铁颗粒,按照先前的文献合成了四氧化三铁纳米粒子^[24]^。简而言之,将FeCl_3_·6H_2_O(1.95 g)、Na_3_C_6_H_5_O_7_·2H_2_O(0.6 g)和乙酸钠(3.6 g)在室温下超声溶解在60 mL乙二醇中。随后,将得到的均匀黄色溶液转移至高压釜中,然后加热至200 ℃保持12 h。反应后产物用磁铁收集,用乙醇和水分别洗涤3次,冷冻干燥备用。

#### 1.3.2 Fe_3_O_4_@TbBd的合成

称取150 mg Fe_3_O_4_NPs、82.91 mg联苯胺和48.6 mg 1,3,5-苯三甲醛于50 mL乙腈中,超声均匀分散后,将2 mL乙酸缓慢加入混合溶液中并进行超声处理,迅速形成黑色沉淀物。静置30 min后用磁铁收集黑色沉淀,用无水四氢呋喃和甲醇交替洗涤6次至上清液澄清。最后,将生成的Fe_3_O_4_@TbBd进行冷冻干燥以备进一步使用^[25]^。

### 1.4 样品采集与处理

在武汉市东湖和长江边分别采集了东湖水样和长江水样,在学校宿舍的洗手池采集了生活废水,将采集好的水样用0.45 μm的亲水滤膜进行过滤,然后放置于4 ℃冰箱中保存以用于后续磁固相萃取分析。

### 1.5 磁固相萃取

所有磁固相萃取实验均在40 mL样品瓶中完成。将15 mg Fe_3_O_4_@TbBd纳米材料分散在10 mL样品溶液中,溶液中每一种对羟基苯甲酸酯的质量浓度为100 μg/L,将上述溶液在室温下超声萃取10 min,然后进行磁分离并弃去上清液。加入1 mL甲醇解吸液,超声解吸10 min,再加入1 mL甲醇重复解吸1次,然后将收集的解吸液用0.22 μm的滤头进行过滤并在40 ℃下用温和的氮气流干燥,最后用100 μL甲醇复溶并进行HPLC-UV分析。

### 1.6 色谱条件

色谱柱为C18反相色谱柱(150 mm×4.6 mm, 5 μm),柱温设置为30 ℃。流动相:A相为乙腈,B相为超纯水;流速设置为1 mL/min。梯度洗脱程序:0~10 min, 35%A~40%A; 10~25 min, 40%A。进样量:20 μL;紫外检测波长设置为254 nm。

## 2 结果与讨论

### 2.1 Fe_3_O_4_@TbBd的表征

通过扫描电镜(SEM)对Fe_3_O_4_纳米粒子和Fe_3_O_4_@TbBd的形貌进行观察(如图2所示)。可以观察到Fe_3_O_4_纳米粒子的分散性较好,呈现出荔枝壳状的粗糙表面,同样Fe_3_O_4_@TbBd也拥有较好的分散性,并且可以观察到在Fe_3_O_4_纳米粒子之间和其球形表层上都存在成片的网状结构,说明材料成功包覆在了Fe_3_O_4_表面。

**图2:**
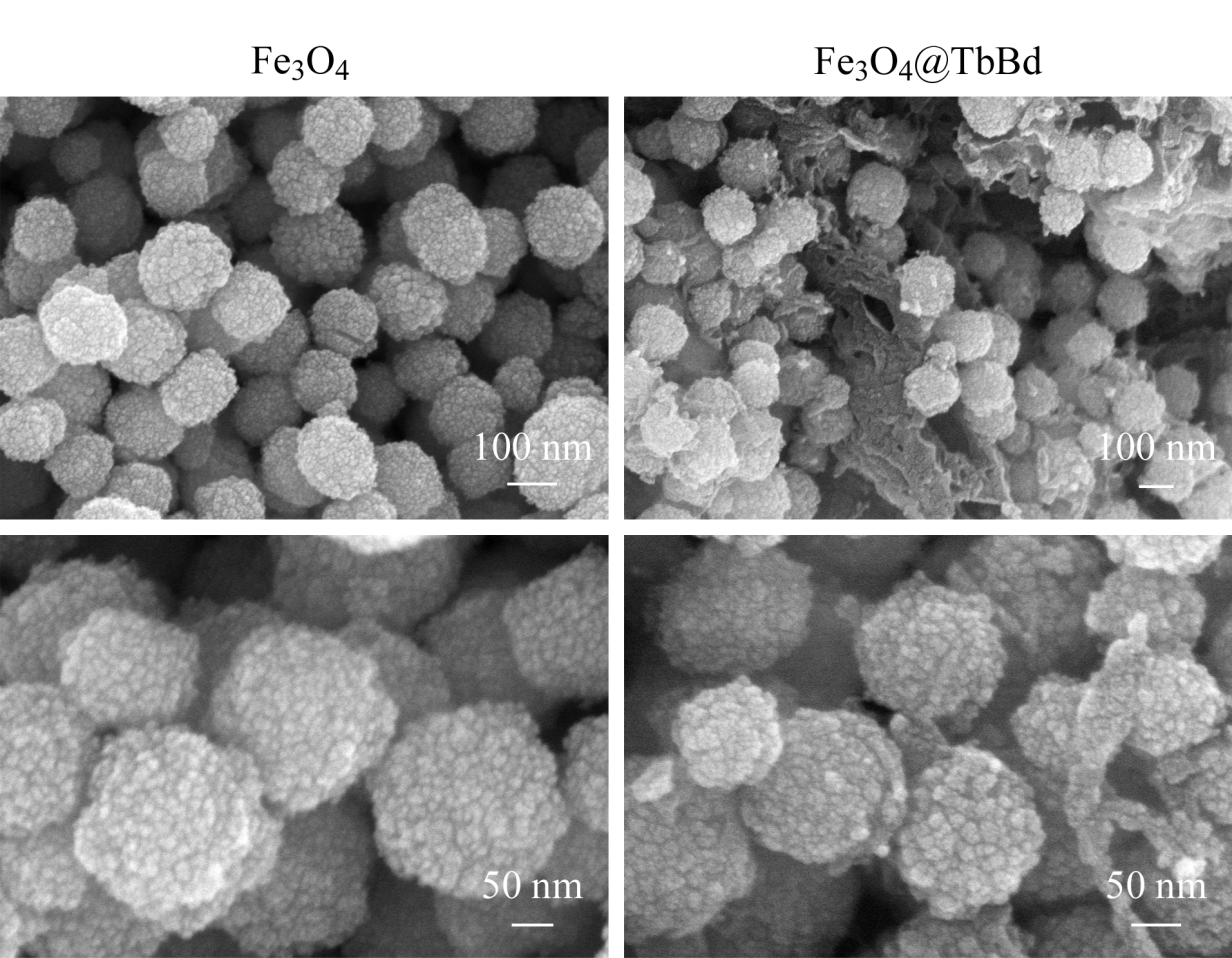
Fe_3_O_4_和Fe_3_O_4_@TbBd的SEM图

通过FT-IR光谱可以证明Fe_3_O_4_@TbBd是否成功合成。从图3a中可以看到,在Fe_3_O_4_NPs的FT-IR光谱中,597 cm^-1^处强红外谱带是Fe-O-Fe的伸缩振动,1404、1612和3428 cm^-1^处的特征吸收峰与羧基的特征峰相符合^[26]^,表明了Fe_3_O_4_表面羧基的存在。包覆在Fe_3_O_4_表面的TbBd是通过氨基单体和醛基单体的席夫碱反应生成的,在Fe_3_O_4_@TbBd的FT-IR光谱中,1417 cm^-1^处出现了C-N键的特征峰,在1611 cm^-1^处有C=N键的伸缩振动^[27]^,证明了COF的成功合成。

**图3:**
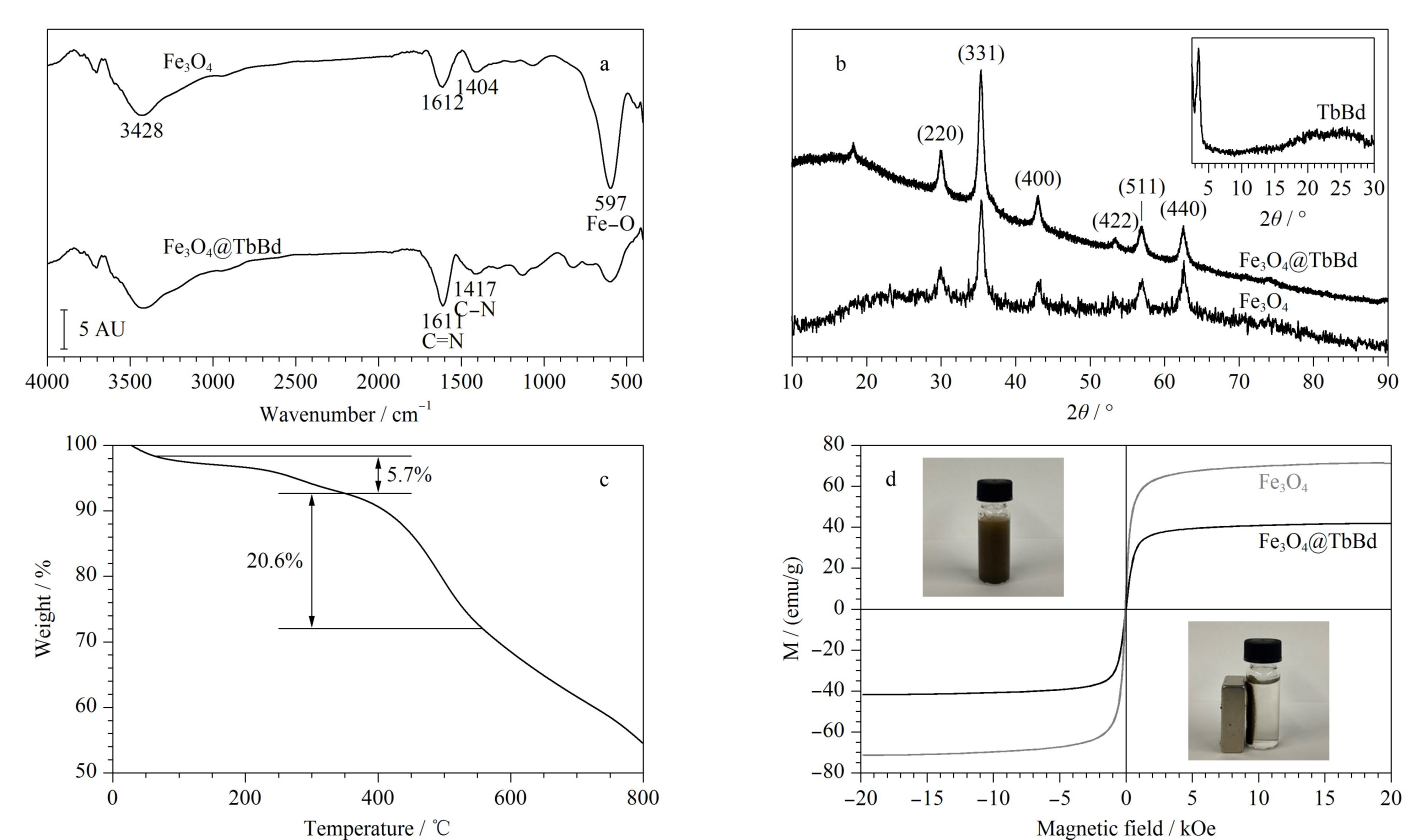
Fe_3_O_4_和Fe_3_O_4_@TbBd的(a)红外光谱图、(b)XRD图、(c)热重曲线和(d)磁滞曲线

图3b显示了Fe_3_O_4_和Fe_3_O_4_@TbBd的X射线衍射(XRD)图。Fe_3_O_4_和Fe_3_O_4_@TbBd在2*θ*=29.9°、35.4°、43.3°、53.0°、57.0°和62.6°处,分别对应(220)、(311)、(400)、(422)、(511)和(440)晶面结构^[25]^。此外,在TbBd的XRD图(如图中插图所示)中,2*θ*=3.51°处有一个小角衍射峰,验证了制备的Fe_3_O_4_@TbBd具有良好的结晶性。

通过热重分析(TGA)来评估Fe_3_O_4_@TbBd的热稳定性,从图3c中可以发现,Fe_3_O_4_@TbBd在350 ℃的范围内有一个缓慢的重量损失,大约为5.7%,这主要为粒子表面物理吸附的水和内部残留溶剂的挥发。而在350~550 ℃内则显示为一个较为急剧的重量损失,约为20.6%,是由于COF材料在高温下分解导致的重量损失。说明材料在350 ℃以下具有良好的热稳定性。

通过振动样品磁强计(VSM)来测定Fe_3_O_4_和Fe_3_O_4_@TbBd的磁性能。如图3d所示,Fe_3_O_4_和Fe_3_O_4_@TbBd的饱和磁化强度值分别为71.3 emu/g和41.7 emu/g,由于在磁性纳米粒子上包覆COF层,使得Fe_3_O_4_@TbBd的磁性降低。如图中插图所示,Fe_3_O_4_@TbBd在水溶液中的分散可在外部磁体的作用下快速聚集(30 s内),使得溶液变得十分澄清。说明Fe_3_O_4_@TbBd具有足够强的磁饱和度用于样品溶液中材料的回收。

### 2.2 MSPE条件优化

采用单因素优化的方法对磁固相萃取过程中MSPE吸附剂用量、萃取时间、溶液pH值、解吸剂种类、解吸时间和解吸次数这些因素进行了优化。在这项工作中,使用10 mL的标准溶液对这些条件进行了考察,该标准溶液含100 μg/L的4种对羟基苯甲酸酯。

#### 2.2.1 吸附剂用量

首先对COF用量在5~20 mg范围内进行研究,其他参数保持不变,吸附时间为30 min,解吸溶剂采用2 mL甲醇,解吸时间为10 min。图4a结果表明,当材料用量从5 mg增加至15 mg时,目标分析物的峰面积逐渐增加,但当继续增加材料的用量至20 mg时,峰面积略有下降,可能是因为用量增加导致材料团聚,其在样品溶液中分散不均匀,COF与目标物相互作用的范围减小,从而导致萃取率降低。因此最终选取材料的用量为15 mg。

**图4:**
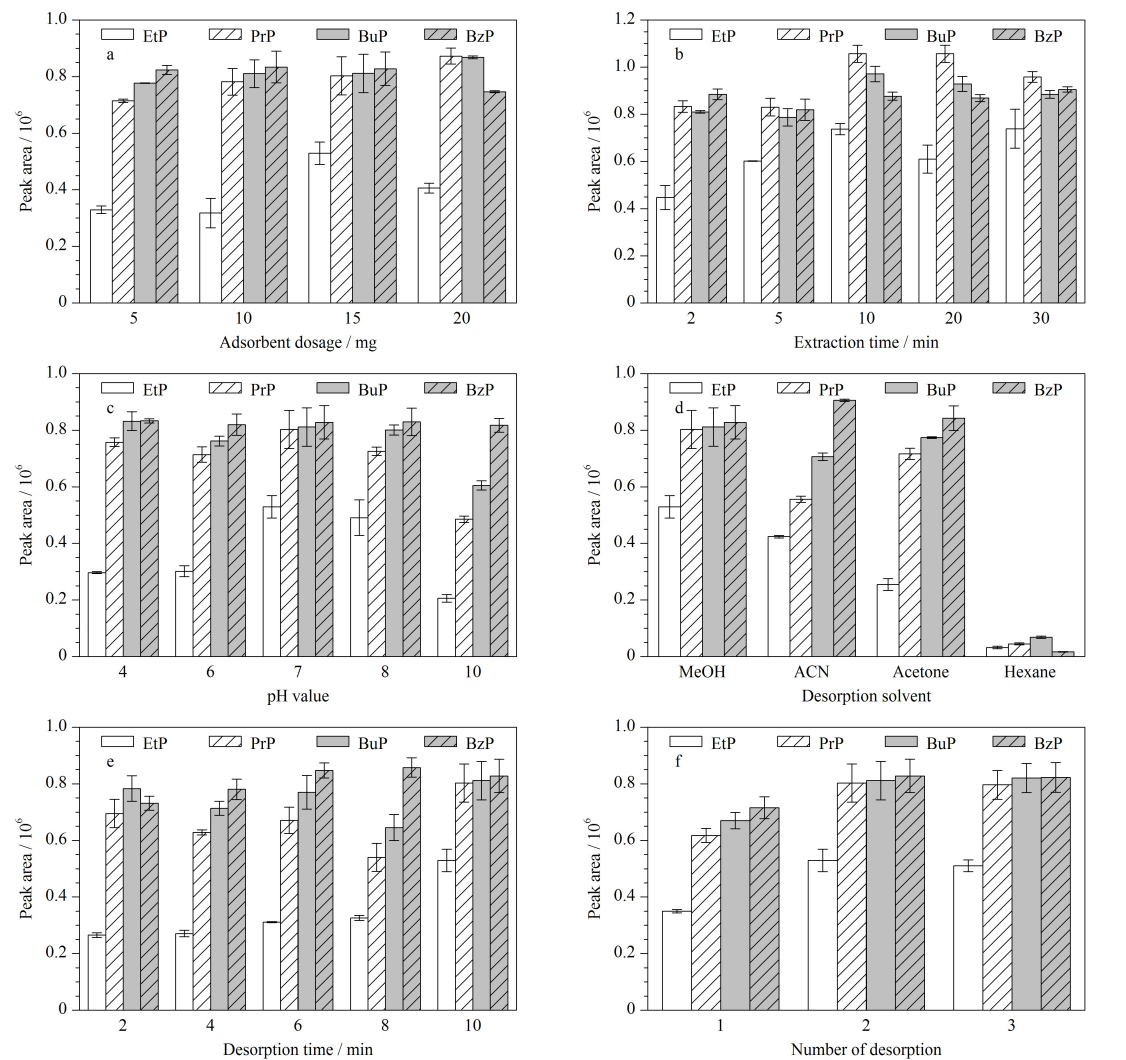
(a)吸附剂用量、(b)萃取时间、(c)pH、(d)解吸溶剂、(e)解吸时间和(f)解吸次数对萃取效果的影响(*n*=3)

#### 2.2.2 萃取时间

进一步优化萃取时间,范围为2~30 min。如图4b所示,4种对羟基苯甲酸酯的峰面积随着萃取时间(2~10 min)的增加而增加,萃取时间从10 min增加至30 min时,峰面积略有下降,但下降幅度很小,可以认为在实验误差范围内整体趋于平衡,因此选择10 min作为萃取时间。

#### 2.2.3 溶液pH值

溶液的pH会影响COF材料的结构和对羟基苯甲酸酯的存在形式,因此在溶液pH为4~10的范围内研究COF材料对对羟基苯甲酸酯的萃取效率。如图4c所示,pH从4增加至8时,4种对羟基苯甲酸酯的峰面积逐渐增加,当pH增加至10时,EtP、PrP和BuP这3种物质的峰面积明显下降,这可能是由于对羟基苯甲酸酯在碱性条件下的离子化,在pH=10时,只有1.8%的目标分析物以分子形式存在,造成萃取效率降低^[28]^。因此,最终选择pH=7的溶液环境进行后续实验。

#### 2.2.4 解吸剂种类

考虑到Fe_3_O_4_@TbBd材料是通过*π*-*π*相互作用对目标物进行吸附的,因此我们按照目标物和解吸溶剂的极性选择了4种不同的解吸溶剂来考察解吸溶剂的影响。为了达到最佳解吸条件,选择了甲醇(MeOH)、乙腈(ACN)、丙酮(ACE)和正己烷(HEX)这4种溶剂作为解吸剂,前3种为极性溶剂,正己烷为非极性溶剂,其峰面积也是最低的,说明极性溶剂更能使对羟基苯甲酸酯从磁性材料上洗脱下来。甲醇和丙酮的极性大于乙腈,实验结果表明(如图4d所示),甲醇和丙酮的效果优于乙腈,由于使用甲醇的洗脱溶液颜色比丙酮洗脱液更加澄清,因此我们选择甲醇作为最佳解吸剂进行后续实验。

#### 2.2.5 解吸时间

解吸时间在2~10 min的范围内进行优化,设置了2、4、6、8、10 min这5个时间点。从图4e中可以看出,解吸时间从2 min增加至10 min时,对羟基苯甲酸乙酯的峰面积也随之增加,其他3种对羟基苯甲酸酯的峰面积没有十分显著的差距,因此选择10 min作为解吸时间。

#### 2.2.6 解吸次数

进行1~3次解吸来优化目标分析物的解吸效率。从图4f中可以看出,解吸次数增加时,4种对羟基苯甲酸酯的峰面积也进一步增加,其中解吸2次和解吸3次的效果基本持平,因此选择2次作为最佳的解吸次数。

### 2.3 方法评价

4种对羟基苯甲酸酯的定量分析通过基于Fe_3_O_4_@TbBd的磁固相萃取结合HPLC-UV进一步评估。在最佳MSPE条件下对4种对羟基苯甲酸酯的线性范围、检出限、定量限、日间和日内精密度及加标回收率进行了评价。实验所得结果如表1所示,4种对羟基苯甲酸酯在其线性范围内线性良好,相关系数(*r*)均大于0.99。根据3倍和10倍信噪比(*S/N*)得到检出限和定量限分别为0.2~0.4 μg/L和0.7~1.4 μg/L。对于日间精密度和日内精密度的考察,从表2可以看出,本方法的日内RSD为1.0%~5.5%(*n*=5),日间RSD为0.1%~4.9%(*n*=3),说明其日内、日间重复性好。低(10 μg/L)、中(50 μg/L)、高(100 μg/L)3种不同水平下得到的加标回收率在86.1%~110.8%之间,说明该方法的准确度良好。

**表1:** 4种对羟基苯甲酸酯的线性范围、相关系数、检出限和定量限

Analyte	Linear range/(μg/L)	Regression equation	r	LOD/(μg/L)	LOQ/(μg/L)
EtP	1-100	y=5637.9x+65309	0.9912	0.2	0.7
PrP	1-100	y=7932.2x+8160.3	0.9991	0.3	0.9
BuP	1-300	y=6882.2x+39552	0.9982	0.4	1.4
BzP	1-500	y=7608.3x+42246	0.9998	0.3	1.1

y: peak area; x: mass concentration, μg/L.

**表2:** 4种对羟基苯甲酸酯的回收率、日内精密度和日间精密度

Analyte	Recoveries/%				Intra-day RSDs (n=5)/%				Inter-day RSDs (n=3)/%					
	10 μg/L	50 μg/L	100 μg/L		10 μg/L	50 μg/L	100 μg/L		10 μg/L	50 μg/L	100 μg/L
EtP	107.9	94.5	93.2		4.1	3.4	4.8		2.2	2.5	3.9
PrP	97.8	103.1	97.7		2.9	5.5	1.0		2.2	3.8	0.1
BuP	92.8	110.8	103.8		3.0	2.5	1.6		1.2	0.7	4.6
BzP	86.1	102.8	103.9		3.4	2.8	2.1		1.2	3.5	4.9

将本方法与近年来其他对羟基苯甲酸酯的分析检测方法进行比较,结果如表3所示。可以看出该方法灵敏度高,与其他样品前处理方法相比,萃取时间短,只需要10 min即可达到萃取平衡,加标回收率处于平均水平,方法重复性良好。因此,该方法适用于环境水样中对羟基苯甲酸酯的分析测定。

**表3:** 本方法与其他方法的比较

Analysis method	Analytes	Samples	Extraction method	Extraction time/min	LOD/ (μg/L)		Recovery/ %		Ref.
HPLC-UV	5 PBs	hand cream	MSPE	30	0.25-	0.34	85.8-	112.6	[29]
HPLC-PDA	4 PBs	cream, sunscreen, etc.	SPE	-	5-	20	85.95-	142.72	[30]
HPLC-DAD	4 PBs	mouthwash, handwash	multi-stir-rod	25	0.63-	0.8	83-	103	[31]
HPLC-DAD	4 PBs	water-based skin toners	MSPE	2	10-	25	98-	106	[32]
HPLC-DAD	4 PBs	scream, lotions	SPME	20	120-	150	90.2-	97.7	[33]
HPLC-UV	4 PBs	beverage, food	MSPE	10	0.51-	1.89	59.2-	109	[34]
HPLC-UV	4 PBs	water, urine	MSPE-DES	8	0.1-	0.3	81.8-	118.2	[35]
HPLC-UV	4 PBs	environment samples	MSPE	10	0.2-	0.4	86.1-	110.8	this work

MSPE: magnetic solid-phase extraction; SPME: solid-phase microextraction; DES: deep eutectic solvent; -: no data.

### 2.4 实际样品分析

将本方法应用于东湖水、长江水和生活废水中对羟基苯甲酸酯的分析,结果如表4所示。在生活废水中检测到了对羟基苯甲酸乙酯和对羟基苯甲酸丙酯这两种物质,含量分别为1.8 μg/L和0.4 μg/L,在东湖水和长江水中均未检测出目标污染物。

**表4:** 实际样品中4种对羟基苯甲酸酯的含量和加标回收率(*n*=3)

Analyte	Added/ (μg/L)	East Lake water				Yangtze water				Domestic wastewater			
		Found/ (μg/L)	Recovery/ %	RSD/ %		Found/ (μg/L)	Recovery/ %	RSD/ %		Found/ (μg/L)	Recovery/ %	RSD/ %
EtP	0	N.D.	-	-		N.D.	-	-		1.8	-	-
	10	10.4±0.7	103.8	3.4		10.2±0.6	102.4	2.9		8.6±1.2	85.7	5.9
	50	46.3±0.1	92.6	0.2		44.5±2.7	88.9	4.0		49.0±1.1	98.0	1.8
	100	94.3±3.0	94.3	2.8		98.2±4.8	98.2	4.4		101.8±4.9	101.86	4.3
PrP	0	N.D.	-	-		N.D.	-	-		N.Q.	-	-
	10	9.7±0.4	96.8	4.0		9.4±0.2	93.8	2.2		11.0±0.1	109.9	1.0
	50	50.9±0.9	101.9	1.8		47.5±1.8	95.6	3.7		56.5±1.8	113.1	1.4
	100	113.6±5.2	113.6	4.5		56.5±0.8	102.8	6.7		101.4±4.5	101.4	4.4
BuP	0	N.D.	-	-		N.D.	-	-		N.D.	-	-
	10	10.1±0.3	101.3	1.9		8.1±1.2	80.7	8.8		8.1±0.6	80.7	4.7
	50	56.5±1.1	112.9	1.7		51.0±1.5	102.0	2.7		58.8±0.5	117.5	0.8
	100	99.5±0.5	99.5	0.5		113.6±5.9	113.6	5.0		92.1±3.4	92.1	3.5
BzP	0	N.D.	-	-		N.D.	-	-		N.D.	-	-
	10	9.5±0.2	95.0	1.5		8.7±1.1	86.7	7.6		10.3±0.6	102.9	3.5
	50	50.8±0.7	101.5	1.3		49.9±0.4	99.8	0.8		57.9±1.9	115.8	2.9
	100	100.8±1.2	100.8	1.1		104.6±5.1	104.6	4.6		103.8±5.4	103.8	4.9

Concentrations of parabens are expressed as average value±standard deviation. N.D.: not detected; -: no data; N.Q.: not quantified but detected.

为了验证方法的准确性,3种样品在低(10 μg/L)、中(50 μg/L)、高(100 μg/L)3种水平下的加标回收率在80.7%~117.5%之间,RSD在0.2%~8.8%之间,说明该方法的准确度与精密度足以满足实际样品中对羟基苯甲酸酯的分析。

## 3 结论

本文通过简单的室温法合成了Fe_3_O_4_@TbBd材料并将其作为磁固相萃取的吸附剂用于萃取4种对羟基苯甲酸酯,材料的合成方便简单,萃取时间短。在此基础上,建立了MSPE-HPLC分析4种对羟基苯甲酸酯的新方法,该方法表现出良好的线性、低检出限、低定量限、高精密度和良好的重复性,能够成功地应用于环境水样中4种对羟基苯甲酸酯的测定。
